# Preliminary Evaluation of the Effect of Body Weight on Contrast Enhancement in Coronary CT Angiography: A Fixed Iodine-Dose Protocol

**DOI:** 10.3390/diagnostics16030368

**Published:** 2026-01-23

**Authors:** Fahad Alraddadi, Hasan Almalki, Rana Saklou, Faris Jawad, Zyad M. Almutlaq, Awad Alzahrani, Meshal Alzahrani, Ghada Alturkstani, Waleed Alharbi, Wed Shaibah, Nasser M. Alzahrani

**Affiliations:** 1 Diagnostic Radiology Department, King Abdullah Medical Complex, Jeddah 23816, Saudi Arabia; 2 Radiological Sciences Department, College of Applied Medical Sciences, King Saud Bin Abdulaziz University of Health Sciences, Riyadh P.O. Box 3660, Saudi Arabia; mutlaqz@ksau-hs.edu.sa; 3 King Abdullah International Medical Research Center, Ministry of National Guard Health Affairs, Riyadh 22384, Saudi Arabia; 4 Radiologic Sciences Department, Faculty of Applied Medical Sciences, King Abdulaziz University, Jeddah, Saudi Arabia; asaalzahrani7@kau.edu.sa (A.A.); mmzahrani@kau.edu.sa (M.A.); 5 Diagnostic Radiology Department, King Fahad General Hospital, Jeddah 23325, Saudi Arabia

**Keywords:** body weight, coronary CT angiography, contrast enhancement, fixed contrast protocol, weight-adjusted protocol

## Abstract

**Objective**: To assess the effectiveness of a fixed contrast injection protocol—75 mL of contrast followed by 40 mL saline at 5 mL/s with an injection duration of 23 s—in achieving diagnostic enhancement in coronary CT angiography (CCTA) using 64-slice detector CT scanner. **Materials and Methods**: 456 consecutive patients with suspected coronary disease who underwent CCTA on a 64-slice detector CT scanner between January 2023 and December 2024 and were retrospectively enrolled. Each patient received 75 mL of contrast medium followed by 40 mL of saline at a flow rate of 5 mL/s, with a total injection duration of 23 s. Two radiologists, blinded to patient information, independently measured the contrast enhancement (HU) values in the coronary segments, ascending and descending aorta, and left ventricle. Attenuation levels ≥250 HU were considered diagnostic. Patients were grouped by body weight into two categories: Group 1 (≤75 kg) and Group 2 (>75 kg). The independent t-test and Mann–Whitney U test were used to compare HU values in each vessel between the two groups, while the Chi-square test was applied to compare enhancement success rates (HU ≥ 250) between the groups per vessel. **Results**: A total of 281 patients (mean age: 51.88 years ± 11.15 [SD]; 167 male, 114 female), were included. Statistically significant differences in the HU enhancement measurements were found between groups (*p* < 0.001–0.007). However, all segments showed mean and median HU values above 250 HU. Enhancement success rates were significantly higher in Group 1 (*p* = 0.005–0.04), except in the ascending aorta, descending aorta, left main coronary artery, middle right coronary, distal right coronary artery, and middle left circumflex artery, where the rates were statistically comparable between the groups (*p* = 0.054–0.61). **Conclusions**: A fixed contrast protocol (75 mL of contrast medium followed by 40 mL of saline at a 5 mL/s flow rate with a total injection duration of 23 s) appears to be feasible for achieving diagnostic contrast enhancement in CCTA using a 64-slice multidetector CT scanner. This protocol may offer a simplified alternative to individualized, weight-based contrast dosing strategies.

## 1. Introduction

In recent decades, advancements in CT technology have led to widespread use of coronary computed tomography angiography (CCTA) as a valuable non-invasive imaging method for evaluating of coronary artery diseases (CAD) [1,2,3]. However, adequate vascular enhancement is essential for accurately detecting and evaluating lesions in the coronary arteries. Previous studies and clinical guidelines suggest that optimal enhancement is achieved when intraluminal attenuation exceeds 250 Hounsfield Units (HU) [4,5]. Therefore, understanding the factors influencing contrast medium injection such as injection rate, volume, and concentration, is critical for achieving consistent vascular enhancement [6]. Body weight and cardiac output are the most important patient-related factors that influence vascular enhancement in CCTA [7].

Body weight has been reported to have an inverse relationship with the degree of vascular contrast enhancement [5]. In CCTA examinations, knowing the scan acquisition duration is crucial for tailoring the contrast medium injection duration and synchronizing CT data acquisition with optimal vascular enhancement [8]. Notably, most of current CCTA studies in the literature either use fixed contrast injection protocols or body weight-adapted contrast injection protocols [9,10,11]. With the advent of newer-generation CT systems and progressively shorter scan durations, the literature increasingly advocates reduced contrast medium volumes in average-sized adult patients [8].

In coronary CTA, both fixed and weight-adjusted contrast injection protocols continue to be employed in routine clinical practice. Although several studies have examined different contrast dosing strategies [9,12,13,14,15,16,17], there remains no consensus regarding an optimal contrast injection protocol that consistently achieves diagnostic image quality with adequate vascular enhancement in CCTA examinations [18], This deficiency in the current literature is particularly evident in studies conducted using 64-detector CT scanners, which, notwithstanding advances in CT technology, continue to be widely used in clinical practice, especially in resource-limited healthcare settings. Additionally, previous studies offered only a limited evaluation of vascular enhancement within the selective cardiovascular vessels [9,12,13]. In contrast, the present study undertakes a more comprehensive assessment of contrast enhancement across multiple coronary arteries and key cardiovascular structures, thereby addressing an important methodological limitation in the existing literature and reinforcing the continued clinical relevance of optimized contrast protocols for widely available CT systems. This study hypothesized that a fixed contrast injection protocol of 75 mL, followed by 40 mL of saline at a flow rate of 5 mL/s, would provide diagnostic enhancement in cardiovascular structures, including the ascending and descending aorta and the left ventricle, and major coronary arteries and their branches across a broad patient weight spectrum in CCTA using a 64-detector CT scanner.

## 2. Material and Methods

### 2.1. Patient Population

This retrospective study was approved by the local ethics committee (No. A01938, 28 May 2024) and conducted in accordance with the Declaration of Helsinki. The informed consent was waived due to retrospective nature of the study. Inclusion criteria included all patients over 18 years old who were referred to the radiology department between January 2023 and December 2024 with suspected CAD and chest pain, and for whom CCTA was performed on a 64-detector CT scanner (Discovery CT 750HD, GE Healthcare, Milwaukee, WI, USA). Exclusion criteria included patients with a history of coronary artery bypass surgery, valve replacement surgery, or previous coronary stent placement, renal insufficiency, and any artifacts affecting the measurement of CT attenuation values (HU), use of an alternative scan protocol, and missing patient weight information.

### 2.2. Imaging Protocol

All examinations were performed on CT scanning on a 64-detector CT scanner (Discovery CT 750HD, GE Healthcare, Milwaukee, WI, USA) using cardiac scanning mode. A prospective ECG-triggered sequence acquisition mode, with the center of the imaging window set at 75% of the R-R interval (padding of 50–100 ms), was used with following parameters: slice collimation (64 × 0.625 mm), scan coverage (40 mm), rotation time (350 ms), tube voltage (120 kV) and tube current (300–650 mA). Images were reconstructed using iterative reconstruction. The field of view was depended on heart size. In patients with a high heart rate (>65 bpm), a beta blocker (50 mg) was given orally to decrease heart rate. Additionally, all patients were received nitroglycerin (5 mg) sublingually for coronary vasodilatation before scanning.

The contrast injection protocol involved injecting 75 mL of iohexol (Omnipaque 350, GE Healthcare, Milwaukee, WI, USA), a 350 mg/mL contrast medium, followed by a 40 mL saline flush at 5 mL/s via an 18-gauge catheter in the antecubital vein. The total injection duration was 23 s; however, this duration represents the total injection time, not the portion of the bolus that is scanned. During the initial phase of the injection, bolus tracking was performed with the ROI placed in the ascending aorta. Image acquisition was triggered once the ROI reached 100 HU, followed by a 3–4 s delay before scanning. The protocol aimed to capture the peak enhancement during the scan duration (10–12 s) (see Supplementary Materials). In patients with normal cardiac output, peak enhancement in the ascending aorta typically occurs at 12–15 s [6], consistent with the timing achieved using this protocol.

### 2.3. Image Analysis

**Figure 1:**
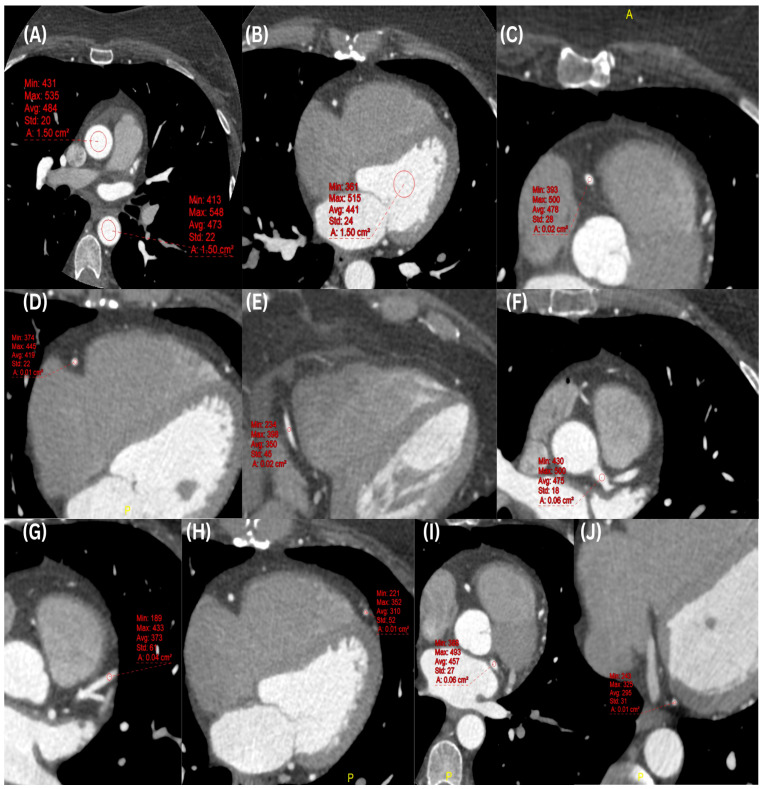
CCTA scan of a 54-year-old female patient with a history of CAD (weight: 96 kg; height: 167 cm). Images (**A**–**J**) display the level of contrast enhancement in the coronary segments and cardiac structures. (**A**) AA = 473 HU, DA = 484 HU; (**B**) LV = 441 HU; (**C**) proximal RCA = 478 HU; (**D**) middle RCA = 419 HU; (**E**) distal RCA = 350 HU; (**F**) LMCA = 475 HU; (**G**) middle LAD = 373 HU; (**H**) distal LAD = 310 HU; (**I**) proximal LCX = 457 HU; (**J**) distal LCX = 295 HU.

Two radiologists with 3 and 5 years of experience in CCTA examinations analyzed the acquired data, blinded to patient identifiers and clinical data, on a dedicated workstation (Centricity™ UV PACS; GE Healthcare, Milwaukee, WI, USA). CT attenuation (HU) was measured by placing the ROIs of 1.5 cm^2^ on axial thin slices (0.625 mm) at the original zoom level in the ascending aorta (AA), descending aorta (DA), and left ventricle (LV). For the coronary arteries measurements —including the left main coronary artery (LM), proximal, middle, and distal segments of the right coronary artery (RCA), left anterior descending artery (LAD), and left circumflex artery (LCX)—ROIs were placed centrally within the lumen and sized proportionally to the vessel diameter to maximize intraluminal coverage while avoiding calcified plaques or stenotic segments. Variable zoom factors (1.0–7.0) were used solely to facilitate accurate ROI placement and did not affect the relative size of the ROI within the vessel lumen (Figure 1). A constant window width of 800 HU and a window level of 100 HU and soft tissue reconstructions were used during all measurements.

The primary outcome was defined by the level of intra-arterial opacification achieved in the coronary arteries, LV, and both AA and DA. Specifically, this study evaluated whether the opacification exceeds the threshold of 250 HU, as this threshold is considered diagnostic adequate enhancement in CCTA with MDCT scanners, as reported by the Society of Cardiovascular Computed Tomography (SCCT) [4]. Opacification measuring HU ≥ 250 was classified as successful, while levels HU < 250 were regarded as suboptimal.

### 2.4. Classification of Patient Groups

Previous studies recommended an iodine-based contrast medium dose of 245–370 mgI/kg [9,19] with a short injection duration for optimal vascular enhancement. At our institution, a fixed contrast bolus of 75 mL is routinely used for all patients undergoing CCTA examinations. Due to 1 mL/kg being widely accepted as the dosing principle required to achieve adequate vascular enhancement [9], a weight threshold of 75 kg was selected to approximate the point at which this standardized bolus provides ≥1 mL/kg of contrast. Patients weighing ≤75 kg therefore received at least 1 mL/kg, whereas those >75 kg received less than 1 mL/kg, which may result in suboptimal vascular enhancement [9,19]. Dividing patients into two groups, Group 1: ≤75 kg; and Group 2: >75 kg, reflects routine practice at our center and would provide a meaningful framework for evaluating whether this simplified, weight-independent protocol achieves diagnostic vascular enhancement across a broad range of body sizes.

### 2.5. Statistical Analysis

Numerical variables (age, body weight, height, and body mass index (BMI) were expressed as means and standard deviations (SDs) for normally distributed data or as medians with interquartile ranges (IQRs) for non-normally distributed data. The independent t-test and Mann–Whitney U test were used to examine differences in contrast enhancement measurements (HU) in each vessel between Group 1 (≤75 kg) and Group 2 (>75 kg). The Chi-square test was employed to compare the success rate of vascular and coronary enhancement (HU ≥ 250) between the two groups in each segment. Statistical analysis was performed using IBM SPSS Statistics version 27. As this is a preliminary exploration study focusing on hypothesis generation, *p*-values for the statistical tests were reported as raw values and were not corrected for multiple comparisons. A *p*-value of less than 0.05 was considered statistically significant.

## 3. Results

**Figure 2:**
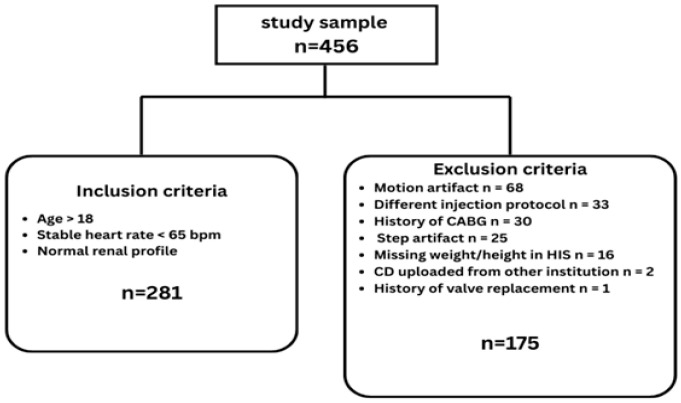
Flow diagram illustrating the initial number of patients and the final number based on inclusion and exclusion criteria for the study.

From January 2023 to December 2024, 456 patients were enrolled at our hospital. A total of 281 patients (mean age: 51.88 ± 11.15 [SD] years; 167 male, 114 female) met the inclusion criteria and were included in the analysis. The study excluded 175 patients for the following reasons: motion artifact (*n* = 68), use of a different injection protocol (*n* = 33), history of coronary artery bypass surgery (*n* = 30), step artifact (*n* = 25), missing weight and height information in the hospital information system on the day of scan (*n* = 16), CD uploaded from another institution (*n* = 2), and history of valve replacement surgery (*n* = 1) (Figure 2).

Based on body weight measurements, Group 1 (≤75 kg) included 138 patients (75 female and 63 male), whereas Group 2 (>75 kg) included 143 patients (39 female and 104 male). The groups demonstrated a statistically significant difference in patient characteristics (Table 1).

### 3.1. Contrast Enhancement Comparison Between Groups

**Figure 3:**
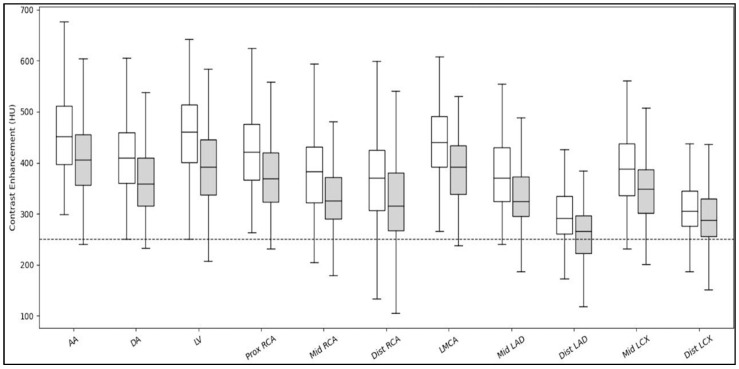
Boxplot of contrast enhancement (HU) between body weight groups across major cardiovascular structures and coronary artery segments. Group 1 (≤75 kg) = white; Group 2 (>75 kg) = gray. AA, Ascending Aorta; DA, Descending Aorta; HU, Hounsfield units; LV, Left Ventricle; RCA, Right Coronary Artery; LMCA, Left Main Coronary Artery; LAD, Left Anterior Descending Artery; LCX, Left Circumflex Artery.

The analysis revealed significant differences between Group 1 (≤75 kg) and Group 2 (>75 kg) in the HU values in each segment. However, all segments demonstrated mean and median HU values exceeding 250 (Table 2, Figure 3).

### 3.2. Success Rate in Each Vessel

There was a significantly higher enhancement success rate in Groups 1 (≤75 kg) compared to Groups 2 (>75 kg) group. However, the AA, DA, middle RCA, distal RCA, LMCA, and Middle LCX were statistically comparable. Additionally, there was a decrease in the success rate in the distal segments of the coronary arteries (Table 3).

## 4. Discussion

An inverse association between body weight and contrast enhancement has been well documented in previous studies [6,7,10]. In alignment with these findings, the present study demonstrated significantly greater contrast enhancement and a higher proportion of successful examinations (attenuation > 250 HU) among patients weighing ≤75 kg compared with those weighing >75 kg. Nevertheless, despite statistically significant differences between the two groups, the fixed contrast protocol (75 mL of contrast medium followed by 40 mL of saline at a flow rate of 5 mL/s) consistently produced an enhancement above 250 HU across all coronary segments, as well as in the ascending and descending aorta and left ventricle. These results indicate that the fixed protocol provides diagnostically adequate vascular enhancement for CCTA using a 64-detector CT scanner.

Bae et al. [10] reported a significant difference in contrast enhancement of the AA between low- and high-BMI groups. However, in contrast to their findings, this study found no significant difference in the success rate of enhancing major vessels, particularly the AA and DA. This discrepancy may be attributed to the scan protocol, which begins at the level of these vessels and permits early image acquisition during peak contrast concentration. Notably, the LMCA also showed no significant variation, which may be attributed to the measurement being performed at the proximal segment, where contrast levels are typically higher. Such factors may have contributed to the comparable enhancement observed across weight groups.

Several factors influence contrast enhancement in CCTA examinations, including contrast-related factors, CT scan–related factors, and patient-related factors. Among the patient-related factors, body weight, heart rate, and age have been identified as key determinants [6,8,20,21]. Body weight has consistently been reported as the most significant patient-related factor influencing contrast enhancement [6], and most institutions design contrast protocols based on patient size [9,13,22]. Several studies have examined the effect of body weight by comparing different body weight–adjusted contrast protocols [12,20,22,23]. A study by Liu et al. [23] reported no significant differences in enhancement between contrast injection protocols. However, patients with a body weight of >95 kg or a BMI of >30 kg/m^2^ were excluded [23]. In contrast, the present study demonstrated a significant difference between groups and included a broader range of body weights (44–130 kg), providing findings that are more reflective of real-world clinical practice.

Modern practices tend to favor weight-adjusted procedures over fixed protocols; however, the wide variation in reported dosing strategies and injection durations has led to considerable uncertainty in clinical practice. For example, Nakaura et al. [9] compared a fixed contrast protocol with a weight-adjusted protocol (1.0 mL/kg) using a short injection duration of 15 s. Their findings indicated that the weight-adjusted protocol provided adequate vascular enhancement while eliminating beam-hardening artifacts commonly observed with fixed protocols [9]. Similarly, Isogai et al. [13] evaluated three different contrast protocols and reported that a dose of 0.7 mL/kg of contrast medium at a concentration of 350 mgI/mL was necessary to achieve optimal enhancement [13]. Another study by Jin et al. [21] demonstrated that a weight-based protocol (0.8 mL/kg) with a 13 s injection duration provided a feasible method for step-and-shoot CCTA, resulting in improved image quality [21]. Nevertheless, results from a recent international survey on CT radiation doses and iodinated contrast media administration revealed a lack of optimization in patient-specific protocols, with significant variability in iodine dosing even within standardized approaches [24]. However, the present study’s fixed contrast protocol achieved average contrast enhancement exceeding 250 HU in all segments across a wide body weight range (44–130 kg) and BMI (14–44 kg/m^2^).

According to Cademartiri et al. [25], high intracoronary attenuation is associated with higher diagnostic accuracy for detecting coronary artery stenosis using MDCT. However, the optimal range of contrast enhancement in coronary arteries has been reported to be 250–400 HU [4,5,7,22]. Fei et al. [26] identified 350 HU as the optimal enhancement level in a study conducted with a specially designed cardiac phantom. They further noted that attenuation above 500 HU could result in underestimation of stenosis and may introduce blooming artifacts [26]. Additionally, the SCCT has recommended values above 250 HU as diagnostically adequate enhancement [4]. The present study therefore adopted ≥250 HU as the criterion for successful enhancement, and the applied fixed contrast protocol achieved enhancement values ranging from 221 to 514 HU, with success rates of 67–100% across all segments and body weight groups.

According to the SCCT guidelines, the contrast medium injection protocol should be adjusted to the scan acquisition duration [4]. The guidelines also suggest a flow rate of 4–7 mL/s to increase the magnitude of aortic enhancement [4]. The present study’s protocol (injection duration of 23 s and flow rate of 5 mL/s) aligns closely with these recommendations and with the recommendation that injection duration should correspond to scan duration, which in this study was 10–12 s, thereby ensuring that peak enhancement was captured during image acquisition.

This study has a number of limitations. First, cardiac output is known to have a significant impact on vascular enhancement in CCTA [6]; it could not be assessed in this retrospective study. Although bolus tracking was used to trigger acquisition at the time of peak aortic enhancement, the absence of cardiac output data remains a limitation and may have contributed to inter-patient variability in attenuation. Second, due to the retrospective design of the study, potential confounders such as heart rate and cardiac output may have affected vascular enhancement. These factors could not be controlled and may, therefore, have contributed to inter-patient variability in attenuation measurements. However, this variability reflects real-world clinical practice where standardization of such parameters is not always feasible. Third, although image quality was not formally evaluated, the fixed contrast dose protocol applied reflects routine CCTA practice at our institution and has consistently yielded diagnostic-quality images without reported concerns from interpreting radiologists. Fourth, low kV settings were not utilized, particularly in the >75 kg group, which may have improved contrast enhancement. Fifth, variation in contrast enhancement was observed, with some segments exceeding 500 HU, a level that may result in underestimation of stenosis due to blooming artifacts [26]. Sixth, body weight classification in this study was determined according to the fixed contrast protocol of 75 mL at 350 mgI/mL. Previous studies have suggested that 1 mL/kg achieves optimal vascular enhancement and falls within the recommended range of 245–370 mgI/kg [9,19]. Therefore, patients in the >75 kg group may have received an adequate iodine dose within the recommended range, corresponding to ≥1 m/kg. Seventh, the lack of BMI-stratified analysis is a limitation of this study, as BMI categories may better capture variations in body habitus. Future study incorporating BMI-based subgrouping may help further refine the application of the fixed-dose protocol. Eighth, radiation dose metrics (CTDIvol and DLP) were not consistently available in this retrospective dataset and, therefore, were not analyzed; the potential interaction between dose settings and contrast optimization remains an important area for future investigation. Ninth, there is a risk of selection bias in this study resulting from the exclusion of a substantial proportion of patients with image artifacts, as such factors would have compromised image quality and reduced the accuracy of CT attenuation measurements. Tenth, the sex distribution was not balanced across weight groups, with a higher proportion of males in the heavier weight category. As sex-related differences in body composition and cardiovascular physiology may influence contrast enhancement, this imbalance represents a potential source of confounding that should be considered when interpreting the findings. Eleventh, multiple statistical comparisons were performed across 11 coronary and vascular segments without adjustment for multiple testing, which may inflate the type I error. However, given the exploratory and hypothesis-generating nature of this preliminary study, the statistically significant findings can be used as a direction for future confirmatory studies. Twelfth, the fixed iodine-dose protocol may lead to variability in contrast enhancement among patients with extreme body weights, which warrants caution when interpreting these findings in broader patient populations. Additionally, this was conducted at a single center using a single CT scanner model, which may limit the generalizability of the findings to other institutions or scanner platforms. Finally, although both radiologists followed a standardized measurement protocol, interobserver variability was not formally assessed, which limits the evaluation of measurement reproducibility.

## 5. Conclusions

The present study demonstrated that a fixed contrast protocol (75 mL of contrast medium followed by 40 mL of saline at a flow rate of 5 mL/s with a total injection duration of 23 s) on a 64-detector CT scanner can serve as an effective alternative to weight-adjusted protocols, with injection duration adapted to scan duration, and may be feasible across a broader body weight range.

## Figures and Tables

**Table 1:** Patient Characteristics.

Variable	Group 1 (≤75 kg)	Group 2 (>75 kg)	*p*-Value
Age	49.70 ± 12.08	52.03 ± 8.84	0.068 **
BMI	26 (24, 28)	31 (29, 34)	<0.001 ***
Height	161 (156, 167.25)	170 (164, 175)	<0.001 ***
Weight	66.565 ± 6.76	90.064 ± 11.93	<0.001 **

** Independent *t*-test. *** Mann–Whitney U test. *p*-values in bold indicate statistical significance (*p* < 0.05).

**Table 2:** Comparison of contrast enhancement (HU) between body weight groups.

Segment	Weight Categories		*p*-Value
	Group 1 (≤75 kg)	Group 2 (>75 kg)	
AA	458.66 ± 81.26	408.05 ± 72.31	<0.001 **
DA	409 (358.75, 460)	359 (315, 410)	<0.001 ***
LV	461 (399, 514)	392 (337, 447)	<0.001 ***
Proximal RCA	424.74 ± 78.09	370.0 ± 70.77	<0.001 **
Middle RCA	382.5 (320.75, 431.25)	326 (290, 372)	<0.001 ***
Distal RCA	372.67 ± 94.13	322.033 ± 89.06	<0.001 **
LMCA	440.5 (391.75, 493.75)	392 (336, 434)	<0.001 ***
Middle LAD	370.5 (324, 431)	324 (295, 374)	<0.001 ***
Distal LAD	291 (261, 335)	266 (221, 298)	<0.001 ***
Middle LCX	387.5 (335, 438.25)	348 (300, 390)	<0.001 ***
Distal LCX	305.5 (273.75, 345)	288 (256, 330)	0.007 ***

AA: Ascending Aorta, DA: Descending Aorta, LV: Left Ventricle, RCA: Right Coronary Artery, LMCA: Left Main Coronary Artery, LAD: Left Anterior Descending Artery, LCX: Left Circumflex Artery. ** Independent *t*-test, *** Mann–Whitney U test. *p*-values in bold indicate statistical significance (*p* < 0.05).

**Table 3:** Success rate of contrast enhancement across body weight groups.

Segment	Weight			*p*-Value
	Group 1 (≤75 kg)		Group 2 (>75 kg)	
AA	n	138	142	0.325
	(%)	100.00%	99.30%	
DA	n	138	140	0.087
	(%)	100.00%	97.90%	
LV	n	138	139	**0.048**
	(%)	100.00%	97.20%	
Proximal RCA	n	138	137	**0.015**
	(%)	100.00%	95.80%	
Middle RCA	n	134	127	**0.007**
	(%)	97.10%	88.80%	
Distal RCA	n	127	121	0.054
	(%)	92.00%	84.60%	
LMCA	n	138	142	0.325
	(%)	100.00%	99.30%	
Middle LAD	n	137	133	**0.007**
	(%)	99.30%	93.00%	
Distal LAD	n	113	96	**0.005**
	(%)	81.90%	67.10%	
Middle LCX	n	136	135	0.61
	(%)	98.60%	94.40%	
Distal LCX	n	124	116	**0.038**
	(%)	89.90%	81.10%	

AA: Ascending Aorta, DA: Descending Aorta, LV: Left Ventricle, RCA: Right Coronary Artery, LMCA: Left Main Coronary Artery, LAD: Left Anterior Descending Artery, LCX: Left Circumflex Artery. *p*-values in bold indicate statistical significance (*p* < 0.05).

## Data Availability

The raw data supporting the conclusions of this article will be made available by the authors on request.

## Supplementary Materials

The following supporting information can be downloaded at: https://www.mdpi.com/article/10.3390/diagnostics16030368/s1, Figure S1. Schematic demonstrating the timing relationship between contrast injection, bolus tracking, trigger threshold, and scan acquisition.
