# Supplementary material for: Preliminary Evaluation of the Effect of Body Weight on Contrast Enhancement in Coronary CT Angiography: A Fixed Iodine-Dose Protocol
Source: Diagnostics (Basel). 2026 Jan 23;16(3):368. doi: 10.3390/diagnostics16030368 (PMC12896695; doi:10.3390/diagnostics16030368)

Supplementary Materials

Figure S1. Schematic demonstrating the timing relationship between contrast injection, bolus tracking, trigger threshold, and scan acquisition

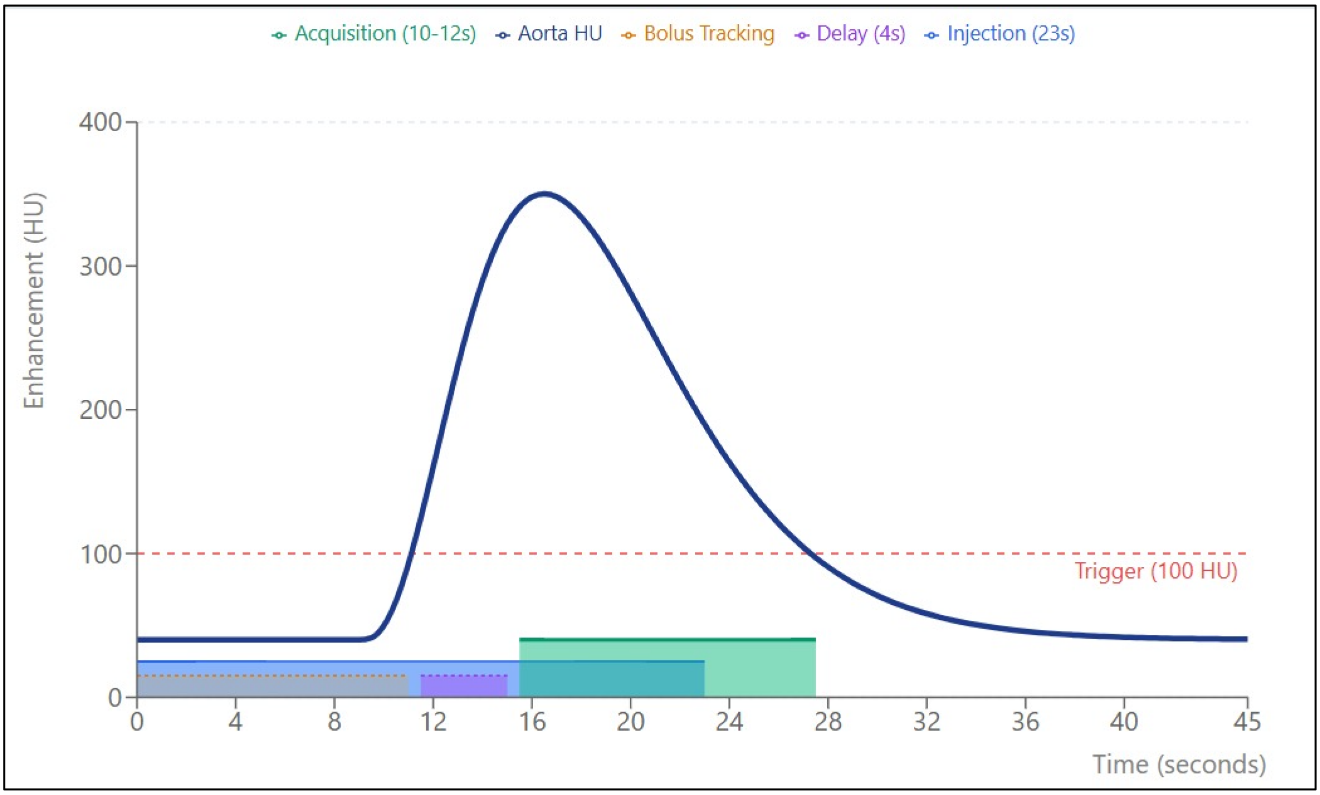

Supplement: Supplementary file 1 [file diagnostics-16-00368-s001.zip › diagnostics-3965224-supplementary.pdf]
